# Ascertainment Bias in a Historic Cohort Study of Residents in an Asbestos Manufacturing Community

**DOI:** 10.3390/ijerph18052211

**Published:** 2021-02-24

**Authors:** Jeremy D. Wortzel, Douglas J. Wiebe, Shabnam Elahi, Atu Agawu, Frances K. Barg, Edward A. Emmett

**Affiliations:** 1 Perelman School of Medicine, University of Pennsylvania, Philadelphia, PA 19104, USA; Jeremy.Wortzel@pennmedicine.upenn.edu (J.D.W.); dwiebe@upenn.edu (D.J.W.); agawua@chop.edu (A.A.); emmetted@pennmedicine.upenn.edu (E.A.E.); 2 School of Medicine, Georgetown University, Washington, DC 20007, USA; se441@georgetown.edu; 3 Department of Pediatrics, Children’s Hospital of Philadelphia, Philadelphia, PA 19104, USA

**Keywords:** historic cohort, asbestos, ascertainment bias, health disparities, asbestos related diseases

## Abstract

This paper describes follow-up for a cohort of 4530 residents living in the asbestos manufacturing community of Ambler, PA, U.S. in 1930. Using re-identified census data, cause and date of death data obtained from the genealogic website Ancestry.com, along with geospatial analysis, we explored relationships among demographic characteristics, occupational, paraoccupational and environmental asbestos exposures. We identified death data for 2430/4530 individuals. Exposure differed significantly according to race, gender, age, and recency of immigration to the U.S. Notably, there was a significant difference in the availability of year of death information for non-white vs. white individuals (odds ratio (OR) = 0.62 *p*-value < 0.001), females (OR = 0.53, *p*-value < 0.001), first-generation immigrants (OR = 0.67, *p*-value = 0.001), second-generation immigrants (OR = 0.31, *p*-value < 0.001) vs. non-immigrants, individuals aged less than 20 (OR = 0.31 *p*-value < 0.001) and individuals aged 20 to 59 (OR = 0.63, *p*-value < 0.001) vs. older individuals. Similarly, the cause of death was less often available for non-white individuals (OR = 0.42, *p*-value <0.001), first-generation immigrants and (OR = 0.71, *p*-value = 0.009), second-generation immigrants (OR = 0.49, *p*-value < 0.001), individuals aged less than 20 (OR = 0.028 *p*-value < 0.001), and individuals aged 20 to 59 (OR = 0.26, *p*-value < 0.001). These results identified ascertainment bias that is important to consider in analyses that investigate occupational, para-occupational and environmental asbestos exposure as risk factors for mortality in this historic cohort. While this study attempts to describe methods for assessing itemized asbestos exposure profiles for a community in 1930 using Ancestry.com and other publicly accessible databases, it also highlights how historic cohort studies likely underestimate the impact of asbestos exposure on vulnerable populations. Future work will aim to assess mortality patterns in this cohort.

## 1. Introduction

Asbestos describes a group of naturally occurring silicate mineral fibers, which have the useful properties of inflammability, poor thermal conductivity, and resistance to corrosive chemicals such as alkalis and acids. As a result, asbestos was used worldwide for the production of fire-resistant materials including asbestos cement, shingles, pipes and many other products. Many studies show a relationship among chronic occupational [1,2,3,4,5,6,7,8,9,10,11,12,13,14] and/or non-occupational [15,16] inhalational asbestos exposure to three major asbestos-induced diseases: lung cancer, malignant mesothelioma (a cancer arising from the pleural or peritoneal lining tissue), and asbestosis (a fibrotic lung disease that can be fatal). These and other conditions caused by asbestos exposure are collectively referred to as asbestos-related diseases (ARD). In the case of lung cancer, smoking and asbestos interact so that asbestos-exposed smokers have a higher than additive risk [17]. Occupational cohort studies defined ARD risks for many industries and occupations including asbestos mining and milling [18], asbestos cement manufacturing [9,19] insulators [20], manufacturing asbestos insulation or textiles [21], and shipbuilding or repair [3,22].

We recognize that there are communities where the risk of ARD is greatly elevated through a combination of occupational and non-occupational exposures [23]. Within these communities, the risk of ARD including mesothelioma might be associated with past occupational, paraoccupational, or environmental (community) asbestos exposures pathways [24]. Paraoccupational exposure [20,25] refers to the exposure of members of the domestic households of occupationally exposed workers through asbestos brought to the home on the workers’ clothes or person [26]. Environmental exposure includes pathways occurring via the location of the residence [25,26] or through lifestyle/behavioral activities in the community [27]. 

In recent years, cohort study designs have attempted to address non-occupational and environmental sources of asbestos exposure. While asbestos actually refers to a class of six naturally occurring silicate minerals, and each type has its own effect on the lung, all types of asbestos are considered dangerous by the US Occupational Safety and Health Administration (OSHA). Studies of paraoccupationally exposed groups have included cohorts of wives of asbestos workers [16]. Cohort studies have demonstrated a link between ARD and environmental exposure experienced by individuals who lived in the crocidolite asbestos mining and milling community of Wittenoom in Western Australia [28,29]; in Libby, MT, where there was mining and milling of asbestos-contaminated vermiculite [8], and in Italian communities where factories produced products containing asbestos [6]. It is important to understand how different exposure pathways influence the risk of ARD. Traditional ARD mitigation efforts have focused on eliminating occupational exposure, but this may be insufficient if significant non-occupational exposure persists. 

Retrospective cohort studies of asbestos-exposed populations contribute to defining the risks of asbestos to health. In these studies, a population with historical asbestos exposure is identified from employment or union records, and subsequent health outcomes are obtained by referring to vital records, other available medical data or by conducting follow-up surveys of individuals. Publicly accessible databases such as the genealogic website, Ancestry.com have been used in a few other studies to follow up on health outcomes of exposed populations, [16,30] but limitations to data ascertainment of these sources have not yet been explored. 

The small town of Ambler, Pennsylvania (PA), U.S., contained the world’s largest manufacturing site for asbestos products from the early 1900s and production continued until 1988. Following the discovery of the insulating properties of a mixture of asbestos fibers and milk of magnesia in 1897, Keasby and Mattison Co. (K&M) produced asbestos-containing sectional pipe coverings and roofing materials in Ambler, an instant market success. To ensure asbestos supply, K&M purchased a large chrysotile mine in Quebec and chrysotile was the predominant fiber used in manufacturing. However, minor amounts of amphiboles were used as well. Major asbestos-containing products manufactured in Ambler from 1900 to 1962 included shingles, piping, electrical insulation, millboard, brake linings, cement siding, paper, conveyer belts, and high-pressure packings [1]. Since K&M’s manufacture preceded federal regulation of waste disposal, dumping of asbestos-containing material (ACM) resulted in large deposits of hazardous waste stock-piled proximate to residential and commercial areas. Since 1984 the Ambler ACM waste sites have been fenced off from the public with warning signage [29,31]. More recently the two major waste areas, called the South Ambler Piles and Bo-Rit sites, have undergone remediation and hazard removal operations as part of the US Environmental Protection Agency (EPA) Superfund program. 

K&M was the largest employer in Ambler and much of the town’s infrastructure and economy was dependent on the company. Therefore, a significant number of Ambler residents were exposed to asbestos through several different pathways including occupational (working in asbestos product manufacturing industry), paraoccupational, and environmental exposure. The Pennsylvania Department of Health, [31] examined cancer incidence reported to the State Cancer Registry from 1992 to 2008 for current residents of the Ambler ZIP code (19002) compared with Pennsylvanian overall. Although the overall cancer rate and the rate of lung cancer were low in ZIP code 19002 relative to the state, the mesothelioma rate was 2.7 times higher than expected in men and 4.5 times higher in women. 

The long induction period for mesothelioma is a major challenge for investigators studying community asbestos exposure. Pooling Italian and Australian data from six occupational exposure and two residential exposure cohorts, Reid et al. [32] found the risk of pleural mesothelioma increased until 45 years after first exposure when it started to plateau, with no one surviving long enough for the excess risk to disappear, while the rate of peritoneal mesothelioma continued to increase for the entire 50 years of study. Lung cancer latency is shorter, with decreasing rates 40 years after exposure ceases [18]. In order to characterize the long-term impact of ARD in the Ambler community we classified exposure and recapitulated demographic data for Ambler in 1930, a time of peak industrial use of asbestos, classifying the principal exposures of residents as occupational, paraoccupational, and environmental using publicly accessible data collection. While future work will include a mortality analysis to assess patterns in the date and cause of death with type of asbestos exposure, in this study we both characterize the multiple exposure pathways of the cohort as well as assess the availability of mortality information to characterize methodological inequities in ascertaining outcomes among different demographic groups within the 1930 cohort. 

Our primary hypothesis is that traditionally marginalized groups, such as African American and first-generation immigrant residents of Ambler, are underrepresented in available mortality records thus underestimating the impact of asbestos exposure among these vulnerable populations, resulting in ascertainment bias. 

## 2. Materials and Methods

### 2.1. Timeframe for Establishing Cohort

We chose the year 1930 for establishing a cohort of Ambler residents based on five major considerations. (1). By 1930, the use of asbestos in manufacturing had been fully developed in Ambler and Ambler established itself as one of the centers of asbestos manufacturing in the U.S. and the world. At this time, asbestos use in production in the US had reached 3849 metric tons per year [33] indicating the national demand for asbestos products and asbestos-containing wastes were present and accumulating on both the South Ambler Piles and Bo-Rit sites. (2). U.S. census data re-identified after 72 years, made individual names, occupation, household occupants, dates and location of birth and other data from the 1930 census publicly available. (3). The choice of 1930 should allow ample time to see ARD outcomes given the very long latency period required to develop lung cancer and mesothelioma. (4). Although many 1930 residents were likely to have subsequently out-migrated, there were still residents living close to the area who could recall conditions around that time and where families had moved, facilitating the subsequent identification of mortality data for former residents. (5). After 1930 there were significant changes in Ambler with the depression leading to bankruptcy and changed ownership of the industrial facilities.

### 2.2. Assembling of the 1930 Ambler Residents Cohort

Through Ancestry.com, a publicly available genealogic database, we accessed records from the U.S. census of all individuals who resided in Ambler in 1930. The records comprised the original information collection forms completed in handwriting during the decennial census through enumeration visits conducted from household to household. The identity of individuals in the U.S. census information become available 70 years after collection. The records were converted into csv data files that were downloaded and used as data. Each row contained the record for one individual including name, address, a household identifier to identify members of a given household, race, age, birth year, birthplace, occupation, immigration status and employer. 

### 2.3. The Exposure Riskscape 

**Figure 1:**
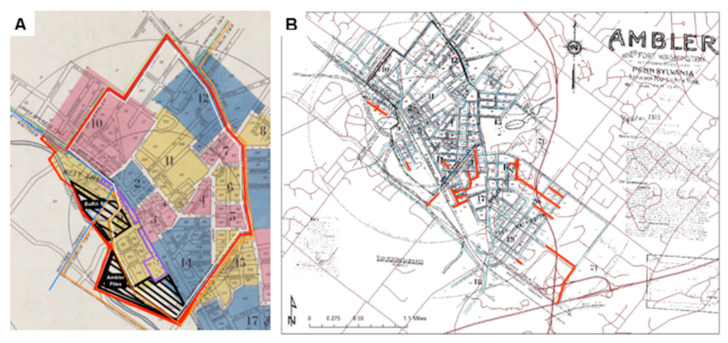
Historic Maps of Ambler, PA (**A**) 1930 Sanborn Fire Insurance Map including Riskscape, asbestos manufacturing, and waste sites superimposed on Sanborn Fire Insurance Map; This map depicts the riskscape enclosed in red, Ambler Borough (white lines), Whitpain Township (blue lines), Upper Dublin (light orange), and Lower Gwynedd (green). The asbestos manufacturing buildings (purple) and waste site areas (black) are also shown. (**B**) Streets that no longer exist in 2016 (indicated in Red), on 1930 Sanborn Fire Insurance map and surrounds.

In order to define an “at-risk population”, we had to first understand the “riskscape” [34], the spatial and temporal distribution of the harmful agent and the pathways through which individuals can be exposed. Previous studies in Ambler used zip code as the relevant boundary for epidemiologic studies of mesothelioma. We sought to examine whether there were alternative ways to conceptualize the boundaries of the exposed population given that the asbestos relationships of interest may not conform geographically to these administratively defined units. Although the three major sites that have contained asbestos fibers or asbestos-containing materials in the Ambler area—the Bo-Rit site, the South Ambler piles, and the former factory—are very close to one another, they are located at the intersections of three distinct governmental entities: Upper Dublin Township, Whitpain Township, and Ambler Borough. Figure 1A shows a historical map of these three locales within the Ambler township. The South Ambler Piles were used as the primary site of asbestos waste contamination for the 1930 cohort as this was the site primarily used throughout the early twentieth century. 

### 2.4. Identifying Potential Community Asbestos Exposure Pathways 

With funding from a Science Education Partnership Award from the National Institute of Health (NIH), (R25 OD010521) and the Center for Excellence in Environmental Toxicology (P30 ES013508), we used a purposive sampling approach to conduct a series of open-ended semi-structured interviews with current and former Ambler residents who had grown up and lived in the area from the 1930s to the present, to identify potential occupational and non-occupational (environmental) exposure pathways. Participants were recruited by a member of the Ambler community. Recruitment continued until the data reached saturation for key themes.

Interviews were conducted by a research coordinator under the direction of the study team during two periods; 2013–2014 and 2015–2016. Interviews were audio recorded with the participants’ permission, transcribed and entered into the qualitative software program NVivo 10.0 for coding and analysis. The study team examined the first five transcripts by conducting a line by line analysis to identify key ideas in the data. These key ideas formed the basis for a codebook. Each code was defined and decision rules for its application were included in the codebook. Codes were applied to all data by two coders from the study team, using the interrater reliability function in NVivo to ensure a minimum of 95% agreement in coding. Codes were summarized, examined for patterns and used to generate key themes related to possible exposure pathways, residents’ perceptions of risk, and insights into life in a community exposed to asbestos. The study was approved by the Institutional Review Board of the University of Pennsylvania (Protocol #817669) We have published a detailed description of the interview study elsewhere [35]. 

### 2.5. Reconstructing and Geocoding the Historic Ambler Riskscape

To determine where each resident resided and may have spent time, we reconstructed the area’s historical geography. The 1930 riskscape boundaries were reconstructed using 1930 Sanborn Fire Insurance Maps for Ambler, accessed from Penn State University Libraries.

Sanborn Fire Insurance maps, originally intended for insurance companies as a way to evaluate fire insurance risks in urban areas, are detailed maps displaying all residential, industrial, and commercial buildings with their corresponding addresses. All house numbers and street addresses within the urban area of the map were listed. Figure 1A displays the Sanborn map for Ambler for 1930. The riskscape for highest asbestos exposure risk was determined considering all four categories of potential exposure pathways: general proximity to the waste piles and factory, location of company-built worker housing, location of schools and recreation areas, and interviewees’ descriptions of the behaviors of children and adolescents. Industrial locations with no residents and no public access were excluded. In Figure 1A this “riskscape” has been superimposed on the Sanborn map. The study area includes not only Ambler borough but also sections of adjacent townships. Almost all of the riskscape was within a 1-mile (1.61 km) radius of the factory site, although the riskscape was not determined by proximity alone. We geocoded by home street address all 1930 Ambler residents who fell within our riskscape of interest. 

### 2.6. Classifying Amber Residents’ Exposure Status

We classified each resident’s likely primary asbestos exposure into one of three exposure pathways: occupational, paraoccupational (asbestos occupation of members of their domestic household), or environmental exposure (community members who could be further classified according to the location of their home and the nature and location of routine daily activities). 

Occupational exposure pathway: occupational exposure to asbestos in Ambler took place in the factory or outside operations between 1897 and 1988. Industry laborers worked for long hours in asbestos polluted air, often with very little or no protective gear, particularly in the early years. Hence, we classified as occupationally exposed those such persons who were listed in the 1930 census as working at the asbestos factory. Secondarily we used census information to classify each occupationally exposed worker as in management or in a labor position. No extant historical occupational information on plant employees was available from the factory at the time of this analysis.

Paraoccupational or household-contact exposure pathway: Paraoccupational or household-contact exposure can be experienced by the family members or others who reside in the same domestic household as those who work with asbestos, as a result of exposure to asbestos from the clothes or bodies of workers bought into the residential environment. Former workers also described bringing home ACM items from the workplace such as asbestos piping which was used at home to cook foods. 

**Figure 2:**
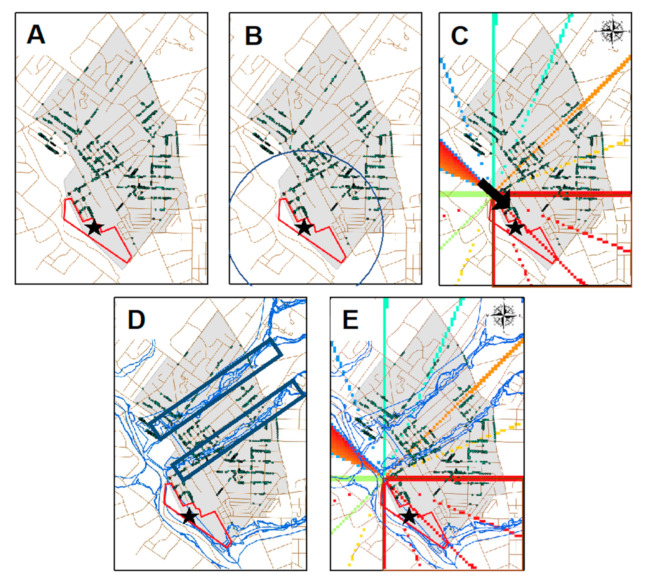
Outline of geospatial methods to identify subjects and classify exposure. (**A**). The digitized Ambler map with the highlighted South Ambler Piles indicated by the red boundary and star, the grey area represents the Ambler township and each green dot represents a member of the cohort. (**B**). A representation of the techniques used in ArcMAP to stratify the cohort by distance. (**C**). A wind-rose established by historic records acquired from the National Ocean and Atmosphere Association was applied to the cohort; a SE prevalence wind was found, thus the SE members of the cohort were classified as downwind of the Ambler Piles. (**D**). The Federal Emergency Management Agency (FEMA) National Flood Hazards Layer within the Ambler township was overlaid and those members of the population within those flood zones were selected. (**E**). A subgroup of individuals (demarked by the magenta area) was selected to represent a portion of the cohort who experienced a concentrated amount of environmental asbestos exposure by being both in close proximity and downwind of the piles.

Environmental exposure pathway profiles: Several steps, graphically summarized in Figure 2, were taken to further define subsets of residents with greater likelihood of community exposure using the geospatial analysis program, ArcMap version 10.8 (ESRI, Redlands, CA, USA) [36]. 

First, the distance between each cohort member’s residence and the South Ambler waste piles was determined. Areas closest to the two waste sites and the K&M factory as well as areas downwind of these sites were assumed to have higher air levels of asbestos [26]. Accordingly, the population that lived closer than average distance (<1852.5 ft) was grouped into a subset which we called ‘Proximity Exposure’ (Figure 2B). We selected 1852.5 ft (0.56 km) distance to assess geographic exposure in part because it encompassed residences of the most vulnerable groups of African Americans and first-generation immigrants and the town of Ambler, identified to have an elevated mesothelioma risk. We did not attempt to extend the boundaries to incorporate all areas of potentially increased ARD risk [37]. Additionally, using this distance as a threshold allowed us to stratify our population in half thereby ensuring an adequate number of residences in each group, and thus adequate power for our study. 

Second, we selected those individuals who were downwind (South-East) of the Ambler Piles to determine a ‘Wind Exposure’ subset. To determine wind patterns, we obtained data from the National Oceanic and Atmospheric Administration, which has made hourly weather readings from a site near Ambler since 1940. Wind direction and speed were used to develop a wind rose for Ambler. Weather data for Ambler exhibit strong temporal autocorrelation, revealing that since 1940 the prevailing wind has consistently been from the west for most of each calendar year. This is also the case for Ambler today. Hence, we are confident in using the wind direction and speed measured during the 1940s to estimate the extent to which cohort members, based on the location of their home, resided downwind in 1930. The wind-rose was applied to our cohort and graphically represented in Figure 2C.

Third, we identified a “Flood Exposure” subset of residents, who lived downstream from the waste piles. This was performed by overlaying FEMA National Flood Hazards data for the Ambler township over the historic maps. Preliminary data and interview data from Ambler residents, support that home flooding may result in higher asbestos exposure and, therefore, was incorporated into this study to further assess its prevalence (Figure 2D). 

Fourth, we grouped the intersection of ‘Proximity Exposure’ and ‘Wind Exposure’ to create a subset of individuals who experienced a concentrated environmental exposure called ‘Proximity and Wind Exposure’ (Figure 2E). ‘Flood exposed’ could be excluded from this grouping as no members of the cohort were exposed to all three environmental parameters. We then broke each environmental exposure profile down by race, gender and immigration status. Independent Chi-Squared analyses were conducted on each subgrouping of the cohort assessing for changes in race, gender and immigration status using the coding language R and R Studio as well as Stata. 

### 2.7. Determination of Date, Age Location and Cause of Death

Death-related records were sought for each resident through publicly available databases including ancestry.com and the National Death Index. Ancestry.com features 727 archives of death-related information that include federal, state, town, cemetery, obituary, church, and other death-related databases. As females may have changed family name after marriage, we also searched marriage records and if found, also searched death records using the married family name. Death record searches used parameters of name, birth year, Ambler, PA, and relevant name of spouse or parents. Matches were considered accurate if the following criteria were consistent with that listed on the 1930 census: full name, birth year within a 3-year range of what was listed on the 1930 census, name of spouse or parents. Additionally, the cohort was stratified into age brackets to better represent the age demographics: children (0–19 y), adult (20–59 y) and older adult (>60 y). These age ranges were chosen to reflect the ages at which residents were likely to live at home with their parents prior to leaving for employment (0–19), be married and/or employed (19–60), or retired (>60). 

**Figure 3:**
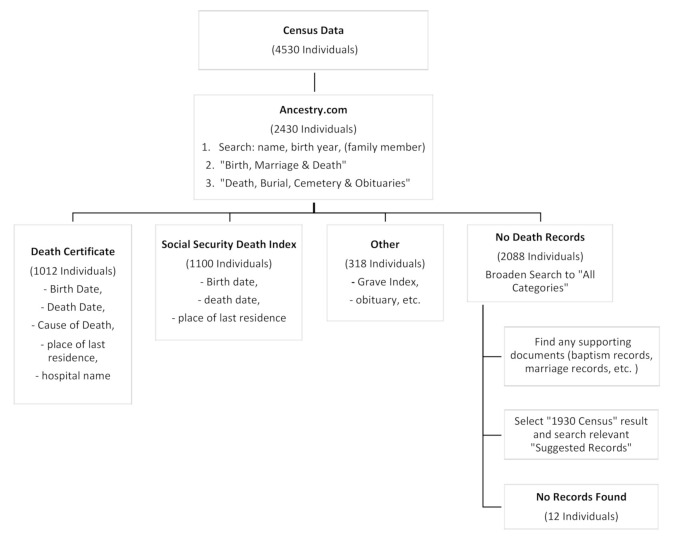
Decision tree for Ancestry.com derived mortality information.

The three-primary death-related databases used from those recorded by Ancestry.com were Pennsylvania Death Certificates, the U.S. Social Security Death Index, and the U.S. Grave Index. Characteristics of these sources are displayed in Table 1. From these sources we collected some or all of the following information: full birth date, full death date, place of last residence, physician, hospital, cause of death, and Social Security number. Less commonly, we used the United States Obituary Collection, Pennsylvania and New Jersey Church and Town records, Pennsylvania Veterans Burial Cards, Pennsylvania Order Sons of Italy in America Mortuary Fund Claims, Connecticut Death Index, and the New Jersey Death Index. The Pennsylvania Order Sons of Italy in America Mortuary Fund Claims database, when relevant, was particularly useful as it included cause of death as well as birth and death dates. Figure 3 displays the decision tree used for obtaining and recording mortality information. Notably, unlike in some countries, death certificates are not generally available from local governments in the U.S.

The National Death Index (NDI) is a US centralized database managed by the Centers for Disease Control (CDC) containing death records drawn from state vital statistics offices. Death information is only available for individuals who died in 1979 or later. We purchased date and cause of death information for individuals in our cohort who could not be identified through the above method. 

## 3. Results

### 3.1. Cohort Demographics and Exposure Subgroups

In our interview study conducted to identify possible mechanisms of exposure, we interviewed past asbestos factory workers (*n* = 7), family members of former asbestos workers (*n* = 15), and residents of Ambler who did not reside in a household with an asbestos worker (*n* = 34). Interviewees ranged in age from 51–83, with the majority (92%) being under 65. Twenty-eight percent of the interview sample was female and 36% was African American. They were recruited for participation by an Ambler community member in collaboration with the study team from the University of Pennsylvania School of Medicine and the Science History Institute. With this approach, we identified potential social, recreational, and geographic pathways of exposure for Ambler residents in addition to previously known occupational and para-occupational routes of exposure.

A total of 4530 individuals were identified as the cohort of residents who lived in the riskscape at the time of the 1930 census in Ambler Borough and West Ambler (collectively designated as Ambler). The mean age of the cohort members was approximately 29 years old with a standard deviation of 20 years. Demographic characteristics and exposures by gender, race and immigration status are presented in Table 2; 474 (10.4%) individuals of the entire cohort were occupationally exposed as workers for K&M, 1638 (36.2%) individuals had para-occupational exposures, 2619 (57.8%) of the population were neither occupationally or paraoccupationally exposed, and 201 (4.44%) were both occupationally and paraoccupationally exposed. Of the entire cohort, 3661 (80.8%) individuals had some form of environmental exposure, including 1365 (30.1%) were experiencing proximity exposure, 2003 (44.2%) were downwind of the Ambler Piles, 62 (1.47%) were both in close proximity and downwind of the Ambler Piles and 231 (5.10%) were within flooding zones. Within the riskscape, approximately 40% of the cohort members resided downwind of the exposure sites, whereas 40% resided to the north and 20% resided to the south of the line of the most direct wind exposure. Additionally, we examined the overlap between occupational, paraoccupational and environmental exposures. Of the occupationally exposed, 38 individuals were also in close proximity to the Ambler Piles and 119 resided downwind. Of those that were paraoccupationally exposed, 132 individuals were also in close proximity to the Ambler Piles and 466 resided downwind. No members of either the occupationally exposed or paraoccupationally exposed cohort were both in close proximity and living downwind of the South Ambler Piles.

Exposures differed by gender, race and immigrant status (Table 2). The factory workforce was predominately male, there were more females in the paraoccupational exposure group. The entire cohort comprised 87.6% whites and 12.4% African Americans. Within the occupationally exposed individuals, African Americans were significantly overrepresented (15.7% versus 9.7% for whites), and in the paraoccupational exposure group at 49.8% versus 29.3% for whites. A similar pattern of higher potential exposure for African Americans compared with whites was seen for the ambient environmental exposure subsets of those living in close proximity, and downwind; however they were less likely to live in flood zones or in residences both in close proximity and downwind of the Ambler Piles. First degree immigrants were significantly overrepresented in both the occupationally exposed and paraoccupational exposure groups compared with second-generation or non-immigrants. First generation immigrants were also more likely than the whole population to be in close proximity, downwind and in flood zones. There was no significant difference between the immigrant status groups for representation in those living both in close proximity and downwind of the Ambler Piles. 

Additionally, when assessing those individuals who had either occupational or paraoccupational as well as environmental exposures, African Americans were statistically more likely to live closer to the South Ambler Piles, while white individuals were more likely to live downwind. Gender disparity also existed when examining these combined exposures as men were more represented in the occupationally exposed individuals who lived in close proximity and downwind, while there was no difference in representation in those paraoccupationally exposed who lived in close proximity and downwind. Finally, first generation immigrants were overrepresented in those who were both occupationally exposed and lived downwind, while those occupationally exposed and in close proximity as well those paraoccupationally exposed in close proximity and downwind had predominantly non-immigrant population.

### 3.2. Characterizing Lifestyle/Behavioral Exposures

Through interviews with community members, numerous individual lifestyle/behavioral activities were associated with potential asbestos exposure pathways including:

- Playing or socializing on asbestos waste piles particularly as a child or adolescent;
- Socialization with asbestos workers (wearing dusty clothing, no hair washing or showering) after work in public venues including bars;
- Picnicking or socializing near asbestos piles as an adult;
- Outdoor sports and gardening
- Using asbestos-containing objects for cooking;
- Flooding into basements with contaminated water from plant or ACM waste sites;
- Eating produce from gardens with contaminated soil.

The interviews identified marked differences in lifestyle/behavioral exposure pathways by age. Playing in the asbestos waste piles during their childhood and adolescence was particularly prevalent at both Bo-Rit and South Ambler Piles and reported by almost all who were children during the period. Activities included playing on the dumpsites, making caves and structures and sledding down slopes of ACM waste, fishing in the reservoir with ACM berms, and other activities. These activities were more frequent for the young who lived closer to the waste-sites but were rarely if ever reported for adults. 

### 3.3. Comparison of Present and Historic Maps

Examination of the 1930 Sanborn map and current maps revealed that many of the 1930 addresses differ from current day listings. Figure 1B illustrates residential streets in 1930 that no longer exist. Most notably Wissahickon Ave, a street containing over 50 row houses built specifically by the K&M to house factory workers and located in the closest proximity to the ACM waste sites, no longer exists because the houses have been demolished and the area incorporated into the expanded waste site. 

### 3.4. Mortality Data Acquisition within the Cohort

**Figure 4:**
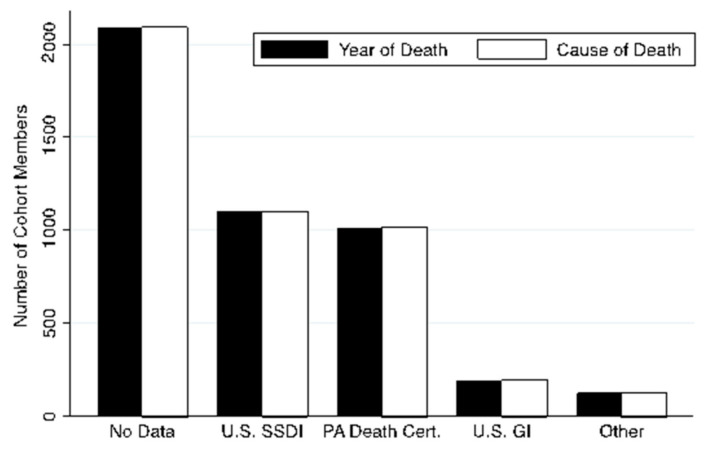
Distribution of information acquisition of death-related databases. U.S. SSDI = United States Social Security Death Index; PA Death Cert = Pennsylvania Death Certificates; U.S. GI = United States Grave Index.

We were able to find records of death for 2430 members (53.6%) of the cohort. The vital status was unknown for the remaining 46.3%. The most useful sources were death certificates obtained through Ancestry.com, the Social Security Death Index and the U.S. Find-a Grave Index (Table 3). Death certificates with cause of death were available for only 22.4% of the cohort. Location of death was available for 1182 of those confirmed as dead (50%). Of these, 413 deaths (34.9%) were reported as occurring while the resident was recorded as living in Ambler. (Table 3) however the location was not specified on 12.3% of Pennsylvania death certificates. Of the three-primary death-related databases used from those recorded by Ancestry.com (including Pennsylvania Death Certificates, the U.S. Social Security Death Index (SSDI), and the U.S. Grave Index), most year of death (yod) and cause of death (cod) information of the 4530 individuals was collected from SSDI (yod and cod: 1100, 24.3%) and the PA Death Certificates (yod and cod: 1012, 22.3%), while only minimal records were collected from the US Grave index (yod and cod: 192, 4.24%) (Figure 4).

### 3.5. Ascertainment Bias within the Cohort

We identified death data for 2430 of 4530 individuals. Ascertainment of year of death differed significantly according to type of asbestos exposure, racial identity and recency of immigration to the U.S. Year of death was less often available for paraoccupationally exposed individuals vs the entire cohort (odds ratio (OR) = 0.59, *p*-value < 0.0001), those living downwind of the South Ambler Piles (OR = 0.64, *p*-value < 0.0001), racial and recency of immigration to the U.S. Year of death was less often available for non-white vs. white individuals (OR = 0.62, *p*-value < 0.0001), females (OR = 0.53, *p*-value < 0.0001), first generation (OR = 0.67, *p*-value = 0.001) and second generation immigrants (OR = 0.31, *p*-value < 0.0001) vs. non-immigrants, and individuals less than age 20 (OR = 0.31 *p*-value < 0.0001) and ages 20 to 59 (OR = 0.63, *p*-value < 0.001) vs. older individuals (>60 y). Similarly, the cause of death was less often available for paraoccupationally exposed individuals vs the entire cohort (OR = 0.41, *p*-value < 0.0001), those living downwind of the South Ambler Piles (OR = 0.02, *p*-value < 0.0001), non-white individuals (OR = 0.42, *p*-value < 0.0001), first generation and (OR = 0.0.71, *p*-value = 0.009) second generation immigrants (OR = 0.49, *p*-value < 0.0001), and individuals less than age 20 (OR = 0.028 *p*-value < 0.0001) and ages 20 to 59 (OR = 0.26, *p*-value < 0.0001). Those living in close proximity were more likely to have records for both year of death (OR = 1.25, *p*-value < 0.0001) and cause of death (OR = 2.37, *p*-value < 0.0001). Occupational exposure did not significantly impact data acquisition of either year of death or cause of death (Table 4).

## 4. Discussion

Using publicly accessible historical U.S. census data, maps and climate data, interviews of long-term community residents and family members to learn about past living circumstances and daily routines, we have been able to characterize patterns of asbestos exposures for residents of a previous generation in a highly contaminated community. We found evidence of significant disparities in likely exposure by race, gender and immigration status, as well as significant disparities in the ability to ascertain date of death.

Our analysis highlights social inequities in asbestos exposure in 1930s Ambler that are likely underestimated due to available methods. Certain trends were observed in this study that reflect the inequitable gender, racial and immigrant dynamics of 1930. The predominance of men in the occupationally exposed cohort and women in the paraoccupationally exposed cohort reflect the gender disparity in the workforce at that time. The statistically significant higher proportions of first-generation immigrants living closer to and downwind of the asbestos piles indicate likely inequity in the town layout and environmental injustice of exposure to asbestos waste. Interestingly, African Americans were less likely to live in close proximity of the major asbestos sources relative to white members of the cohort, but more likely to live downwind, whereas first generation immigrants were more likely than the general community to be both in close proximity and downwind of the waste sites. This indicated that other factors, beyond minority status, such as residential segregation may have played a role in where members of the community resided. This evidence aligns with our understanding of the Ambler factory town layout where factory owners built houses for workers that were right up against the plant, but residential segregation of the time may have excluded African Americans from living in those houses.

Noonan et al. [27] characterized and roughly quantified environmental exposure pathways in Libby Montana over the past few decades. Unfortunately, there are no measurements of environmental asbestos exposures from Ambler around the 1930s that allow any useful exposure quantification. However, it is informative to compare the types of environmental exposure experienced by 1930s residents of Ambler and those reported for Libby. Although there are broad parallels, lifestyle and recreational exposures are rather community specific and strongly influenced by local behavior patterns. Significant potential Libby exposures were reported from cutting firewood (with asbestos contamination of bark) and burning that wood at home, hiking and biking along contaminated roads, heating asbestos-contaminated vermiculite ore to make it “pop”, handling ore outside the job, and from fishing. Ambler residents reported exposures from flooding of houses, community contacts with factory workers socially after shifts, socializing or playing on or near asbestos waste piles, and cooking on asbestos containing objects. Some exposures were common to each group such as through gardening and playing sports on contaminated grounds. Similar to Noonan et al. [27], we identified children and adolescents at risk from behavior patterns specific to those groups. We suggest that particular attention needs to be given in contaminated communities to avoiding such behaviors as far as possible. Usual practices of fencing and containment are unlikely to be sufficient for adolescents.

This study also utilized several methodologies in innovative ways to best assess the asbestos exposure experienced by this community. Sanborn Fire Insurance maps have previously been used to understand physical changes in urban landscapes [32,38] as well as the spread of historical epidemics [36]. However, Sanborn Fire Insurance maps of Ambler from 1930 compared with the present topography demonstrate the need to reconstruct the past residential riskscape to follow the population at risk forward. Without the use of historical maps, residences from 1930 that at the time were closest to the factories and waste area that likely posed the greatest risk would not have been identifiable. Additionally, while researchers have previously used the Ancestry.com Social Security Death Index as an inexpensive tool to collect basic mortality or clinically related information [30,39,40], we extended that methodology search to all the death databases available through Ancestry.com. Doing so allowed us to identify mortality information for over half the population and to access death certificates which include the cause and location of death for around one-half of these individuals. The use of Ancestry.com data both for cohort identification through the historic census data, as well as ascertainment of mortality is a unique opportunity in the study of diseases with long latency periods such as ARD. We hope that this approach can provide added benefits to future studies assessing similar diseases with long latency times.

Understanding the true health impact of exposure to asbestos is hampered by a long latency period, multiple exposure pathways, difficulty in measuring past exposures, incomplete population data, migration of exposed individuals, and inexact methods of recording cause of death. These factors may account for the finding in a recent Pennsylvania Department of Health analysis of Cancer Registry data that Ambler had a significant excess incidence of mesothelioma in both men and women but no increase in lung cancer. An increase in both cancers might have been expected based on findings in heavily exposed occupational cohorts. This may reflect the particularly long latent period for mesothelioma, smoking rates of the population, and the lower levels of exposure such as are encountered with para-occupational and environmental pathways [41]. However, insufficient information was available to understand the ratio of mesothelioma to lung cancers rates for Ambler from the 1930 period. However, because adverse outcomes from exposure continue to the present day, it is necessary to profile individuals and groups who are most at risk. We have identified that race, age, gender and immigration status are likely important risk factors. However, these results suggest that ascertainment bias is important to consider in analyses that investigate occupational, para-occupational and environmental asbestos exposure as risk factors for mortality in this and other historic cohorts. Historic cohort studies likely underestimate the impact of asbestos exposure on more vulnerable populations.

This study approach has limitations. From the perspective of measuring outcomes, it is a logistical challenge to identify death information for each member of a cohort who was alive in 1930 and, therefore, losses to follow up and lack of death information were both major limitations of this study. As we described, Ancestry.com turns out to be a resource that is highly valuable for this goal. Moreover, given that much of information accessed by Ancestory.com is composed of publicly available data online and those databases are being updated continually, we expect this method will become even more useful in the future. Nevertheless, significant ascertainment bias indicated that certain populations such as non-whites, females, recent immigrants and younger individuals were less represented in the obtained death data. Additionally, none of the databases that were assessed provided information to corroborate interviewee descriptions of lifestyle and behavior related to asbestos exposure that may have led to an underestimation asbestos exposure in the cohort. Furthermore, no extant historical occupational information was available which severely limited the ability to better stratify occupational exposure as this may vary substantially. From the perspective of measuring exposure, using the 1930 cohort and classifying exposure as we have attempted to estimate exposure in multiple pathways, but at only a single point in time. A planned next step is to access decennial census data for Ambler earlier (1920) and later, including 1950, as available, in order to identify to the extent possible whether the exposure statue of each cohort member changed or stayed the same. Doing so achieves a way to gauge essentially dose information, that is, information on the length of time over which individuals evidently were exposed to asbestos though different pathways. Finally, while mortality records have been found for some members of the cohort, there is difficulty in accounting for the major latency of mesothelioma in clinical presentation. While this study focused on these methodological challenges of data collection, current efforts are underway to classify mortality into larger groups to assess how mortality and the exposure profiles we itemized in this paper relate to one another.

Despite these challenges, the strengths of a cohort study for asbestos-related disease include: (1) inclusion of all persons exposed to the hazard at the time the cohort was established; (2) minimization of effects of lost cases due to subsequent out-migration from the area; (3) resilience against in-migration with dilution of any potential effect by persons who did not sustain historical exposures; (4) robustness against subsequent changes in the geographic riskscape such as redevelopment of old neighborhoods; (5) relative independence from the difficulties in retrospective ascertainment of past personal exposure, particularly from spouses or next-of-kin lacking first-hand knowledge of circumstances many years previously. Cohort studies also have potential value in enabling the study of interactions between social and environmental factors operating during the time of exposure. We hope the current study inspires new investigations into exposure-related harm in other communities where this issue has been historically challenging to study.

## 5. Conclusions

To address the challenges of studying the role of different asbestos exposure pathways in Ambler, Pennsylvania, we instituted a historical cohort study of residents of the town in 1930 using publicly accessible databases. We have outlined the methods used to classify individuals as occupationally, paraoccupationally and environmentally exposed to asbestos. We found socioeconomic factors such as gender, race and immigration status corresponded to occupational, paraoccupational and some forms of environmental asbestos exposure. Additionally, we found that adolescents sustained a unique set of community and behavioral exposures relative to other age groups. We used a holistic approach, whereby the analysis deliberately considered how these different and potentially overlapping routes of exposures impact an entire community. Finally, significant ascertainment bias of death data discovered in our analysis highlights the limitations of describing the true impact of these exposure pathways on vulnerable populations, a continued challenge to the goal of achieving environmental justice.

## Figures and Tables

**Table 1:** Description of Ancestry.com databases as sources of death information.

Database Title	Location	Dates	Data	Source
PA, Death Certificates	PA	1906–1963 Note: 1925 is missing from the archive	- Name and residence of the decedent - City and county of death - Gender and race - Marital status - Age and date of birth - Occupation - Place of birth - Parents’ names and birthplaces - Date of death - Dates attended by physician and address - Length of stay in hospital or institution - Length of residence for recent arrivals - Place of burial or removal - Date of burial - Undertaker name and address - Name and address of informant	Pennsylvania Department of Health
U.S. Social Security Death Index	U.S.	1935–2011 Note: Social Security number is not available for those who passed within the past 10 years.	- Last name - First name - Social Security Number - State issued - Birth date - Death date - Last residence - Lump sum payment	Social Security Administration, Social Security Death Index, Master File, Social Security Administration
U.S. Find A Grave Index	U.S.	1600s–Current	- Last name - First name - Birth date - (additional data varies by user)	All data is uploaded by Find-A-Grave volunteers to http://www.findagrave.com

**Table 2:** Itemized exposure profile by race, gender and immigration status.

**2A**		**(*n*)**	**Entire Cohort** **(*n* = 4530)**	**(*n*)**	**Occupational Exp. (*n* = 474)**	**sig**	**(*n*)**	**Para-occupational Exp. (*n* = 1638)**	**sig**	**(*n*)**	**No Occupational or Paraoccupational Exp** **(*n* = 2619)**	**sig**	**(*n*)**	**Occupational & Paraoccupational Exp. (*n* = 201)**	**sig**
**Avg Age in 1930 +/− sd**		4530	29.65 +/− 20.29	474	38.5 +/− 14.5	**	1638	24.6 +/− 18.8	**	2619	31.7 +/− 21.0	**	201	35.5 +/− 14.9	**
**Avg Dist (ft) from Waste Piles +/− sd**		4530	1852.5 +/− 1159.7	474	1574 +/− 1124.6	**	1638	1560 +/− 1124.6	**	2619	2183.6 +/− 1067.6	**	201	1490.0 +/− 1061.1	**
**Race**	**% black**	560	12.39	88	18.57	**	321	19.6	**	205	35.8	**	42	20.9	**
	**% white**	3958	87.61	386	81.43		1317	80.4		2414	61		159	79.1	
	**% other**														
	**total**	4518	100	474	100		1638	100		2619	100		201	100	
**Sex**	**% male**	2232	49.38	419	88.4	**	768	46.89	**	1268	55.4	*	167	83.1	**
	**% female**	2288	50.62	55	11.6		870	53.11		1341	60.1		34	16.92	
	**total**	4520	100	419	100		1638	100		2609	100		201	100	
**Img. Stat.**	**% 1st Gen**	712	15.74	195	41.14	**	314	19.17	**	282	39.6	**	79	39.3	**
	**% 2nd Gen**	857	18.94	37	7.81		446	27.23		393	45.9		19	9.45	
	**% Non-Immigrant**	2955	65.32	242	51.05		878	53.6		1939	65.6		103	51.2	
**2B**		**(*n*)**	**Proximity Exposure**	**sig**	**(*n*)**	**Wind Exposure** **(*n* = 2003)**			**sig**	**(*n*)**	**Proximity and Wind Exposure** **(*n* = 62)**	**sig**	**(*n*)**	**Flood Exposure** **(*n* = 231)**	**sig**
			**(*n* = 1365)**												
**Avg Age in 1930 +/− sd**		1365	30.0 +/− 20.8	*	2003	24.9 +/− 18.3			**	62	29.8 +/− 18.8		231	25.3 +/− 18.5	*
**Avg Dist (ft)from Waste Piles +/− sd**		1365	1232.4 +/− 619.6	**	2003	534.3 +/− 563.3			**	62	983.1 +/− 673	**	231	1836.7 +/− 662.9	
**Race**	**% black**	68	4.9	**	216	28.6			**	8	5.41	*	17	7.4	**
	**% white**	1296	94.9		540	71.4				140	94.6		213	92.2	
	**% other**	1	0.073												
	**total**	1365	99.873		756	100				61	100		230	99.6	
**Sex**	**% male**	669	49		408	54			*	74	50		123	53.2	
	**% female**	696	51		348	46				74	50		108	46.8	
	**total**	1365	100		756	100				62	100		231	100	
**Img. Stat.**	**% 1st Gen**	241	17.7	**	154	20.4			**	24	16.2		64	27.7	**
	**% 2nd Gen**	291	21.3		213	28.2				37	25		82	35.5	
	**% Non-Immigrant**	833	61		389	51.5				87	58.8		85	36.8	
**2C**		**(*n*)**	**Occupational & Proximity Exp.** **(*n* = 38)**	**sig**	**(*n*)**	**Occupational & Wind Exposure (*n* = 119)**			**sig**	**(*n*)**	**Paraoccupational & Proximity Exposure** **(*n* = 132)**	**sig**	**(*n*)**	**Paraoccupational & Wind Exposure** **(*n* = 466)**	**sig**
**Avg Age +/− sd**		38	36.1 +/− 13.7	*	119	35.9 +/− 12.2			**	132	28.7 +/− 18.2		466	20.9 +/− 16.8	**
**Avg Dist (ft)from Waste Piles +/− sd**		38	3675.628 +/− 285.4	**	119	358.6 +/− 366.2			**	132	3906.7 +/− 1081.5	**	466	347.4 +/− 328.7	**
**Race**	**% black**	24	63.2	**	36	30.25			**	86	65.2	**	148	31.8	**
	**% white**	14	36.8		83	69.75				46	34.9		318	68.2	
	**% other**														
	**total**	38	100		119	100				132	100		466	100	
**Sex**	**% male**	35	92.1	**	108	90.8			**	59	44.7		231	49.6	
	**% female**	3	7.9		11	9.24				73	55.3		235	50.43	
	**total**	38	100		119	100				132	100		466	100	
**Img. Stat.**	**% 1st Gen**	2	5.26	*	63	52.9			**	6	4.55	**	94	20.2	**
	**% 2nd Gen**	0	0		10	8.4				3	2.27		168	36.1	
	**% Non-Immigrant**	36	94.7		46	38.7				123	93.2		204	43.8	

(**2A**). Itemized occupational and paraoccupational exposure profile; (**2B**). Itemized environmental exposure profile; (**2C**). Itemized combined exposure profile. Img. Stat. = Immigration Status; Proximity exposure: cohort living ≤ 1852.5 ft (average distance if the cohort) from the Ambler Piles; Wind exposure: cohort living downwind (SE) of the Ambler Piles; Flood exposure: cohort who lived with in flood zones. Percent (%) race, gender and immigration status conducted using the total population of the subgroup being analyzed using the number (*n*). Student *t*-tests and chi-squared tests were conducted. (*) for *p*-value < 0.05 and (**) for *p*-value < 0.0001.

**Table 3:** Availability and location of death data from Ancestry.com from 1930 Ambler residence.

		Frequency (%)
**Death Data Source**	Death Certificate	1014 (22.4)
	Social Security Death Index	1046 (23.1)
	US Find-a-Grave Index	189 (4.2)
	Other	132 (2.9)
	No data found *	2139 (47.3)
Death Certificate Source **	Pennsylvania	992 (97.8)
	Philadelphia	13 (1.3)
	North Carolina	2 (0.2)
	Texas	2 (0.2)
	Vermont	1 (0.1)
	Ohio	1 (0.1)
	Florida	3 (0.3)
**Location of Death** (**if known**)	Pennsylvania	1005 (83.2)
	Outside of Pennsylvania	177 (16.8)
	Ambler ⍭	413 (41.1)
	Outside of Ambler ⍭	447 (44.5)
	Not Indicated ⍭	145 (14.4)

(*) Indicates vital status unknown; (**) indicated those with a death certificate on Ancestry.com; (⍭) indicates those who died in Pennsylvania.

**Table 4:** Ascertainment of death data in 1930s Ambler cohort.

	YOD Present (*n* = 2452) N (%) ^1^	YOD Absent (*n* = 2078) N (%) ^1^	OR (95% CI) ^2^	*p*-Value	COD Present (*n* = 567) N (%) ^1^	COD Absent (*n* = 3963) N (%) ^1^	OR (95% CI) ^2^	*p*-Value
**Occupational Exposure**	260 (54.9)	214 (45.2)	1.02 (0.85, 1.24)	0.78	65 (13.7)	409 (86.3)	1.12 (0.85, 1.48)	0.42
**Para- occupational Exposure**	754 (46.0)	884 (54.0)	0.59 (0.53, 0.67)	<0.0001 **	115 (7.0)	1523 (93.0)	0.41 (0.33, 0.50)	<0.0001 **
**Proximity Exposed**	337 (58.7)	237 (41.3)	1.25 (1.04, 1.49)	0.014 *	122 (21.3)	452 (78.8)	2.37 (1.89, 2.97)	<0.0001 **
**Wind Exposed**	340 (45.0)	416 (55.0)	0.64 (0.55, 0.75)	<0.0001 **	25 (3.32)	731 (96.7)	0.20 (0.14, 0.31)	<0.0001 **
**Black**	250 (43.71)	322 (56.29)	0.62 (0.52, 0.74)	<0.0001 **	35 (6.12)	537 (93.88)	0.42 (0.29, 0.60)	<0.0001 **
**Female**	1035 (46.37)	1197 (53.63)	0.53 (0.47, 0.60)	<0.0001 **	262 (11.74)	1970 (88.26)	0.86 (0.72, 1.03)	0.106
**1st Gen Immigrant**	343 (48.17)	369 (51.83)	0.67 (0.57, 0.79)	0.001 *	76 (10.67)	636 (89.33)	0.71 (0.55, 0.92)	0.009 *
**2nd Gen Immigrant**	388 (45.27)	469 (54.73)	0.59 (0.51, 0.69)	<0.0001 **	65 (7.58)	792 (92.42)	0.49 (0.37, 0.64)	<0.0001 **
**Young** (**≤19 y**)	679 (42.41)	922 (57.59)	0.31 (0.25, 0.39)	<0.0001 **	30 (1.87)	1571 (98.13)	0.028 (0.019, 0.042)	<0.0001 **
**Middle-Age** (**20–59 y**)	1445 (59.83)	970 (40.17)	0.63 (0.51, 0.79)	<0.0001 **	363 (15.03)	2052 (84.97)	0.26 (0.21, 0.32)	<0.0001 **

Table 4 Legend: Death data obtained included year of death (YOD) and cause of death (COD). Data collected for 1st and 2nd generation immigrants were compared to the data obtained for non-immigrants, and young and middle-aged individuals were compared to the data available for older cohort members (>60 y). (^1^) Chi-squared contingency; Test, (^2^) Logistic regression analysis; (**) for *p*-value < 0.0001; (*) for *p*-value < 0.05.

## Data Availability

The qualitative interview data are not publicly available due to privacy concerns. The quantitative data presented in this study are available from the corresponding author. The data are not publicly available due to privacy concerns.

## Abbreviations

Keasby & Mattison Co. (K&M); Environmental Protection Agency (EPA); Asbestos related diseases (ARD).
